# Genome-Wide Characterization and Functional Validation of the ACS Gene Family in the Chestnut Reveals Its Regulatory Role in Ovule Development

**DOI:** 10.3390/ijms25084454

**Published:** 2024-04-18

**Authors:** Yanhong Cui, Xingzhou Ji, Wenjie Yu, Yang Liu, Qian Bai, Shuchai Su

**Affiliations:** 1 College of Forestry, Beijing Forestry University, Beijing 100083, China; cuiyh@bjfu.edu.cn (Y.C.); jxz2000@bjfu.edu.cn (X.J.); buayuwenjie@163.com (W.Y.); bualiuyang@163.com (Y.L.); 2 State Key Laboratory of Efficient Production of Forest Resources, Beijing 100083, China; 3 Beijing Advanced Innovation Center for Tree Breeding by Molecular Design, College of Plant Science and Technology, Beijing University of Agriculture, Beijing 102206, China

**Keywords:** *Castanea mollissima*, ACS gene family, genome-wide analysis, expression patterns, ovule development, genetic transformation

## Abstract

Ovule abortion significantly contributes to a reduction in chestnut yield. Therefore, an examination of the mechanisms underlying ovule abortion is crucial for increasing chestnut yield. In our previous study, we conducted a comprehensive multiomic analysis of fertile and abortive ovules and found that *ACS* genes in chestnuts (*CmACS*) play a crucial role in ovule development. Therefore, to further study the function of *ACS* genes, a total of seven *CmACS* members were identified, their gene structures, conserved structural domains, evolutionary trees, chromosomal localization, and promoter cis-acting elements were analyzed, and their subcellular localization was predicted and verified. The spatiotemporal specificity of the expression of the seven *CmACS* genes was confirmed via qRT–PCR analysis. Notably, *CmACS7* was exclusively expressed in the floral organs, and its expression peaked during fertilization and decreased after fertilization. The ACC levels remained consistently greater in fertile ovules than in abortive ovules. The ACSase activity of *CmACS7* was identified using the genetic transformation of chestnut healing tissue. Micro *Solanum lycopersicum* plants overexpressing *CmACS7* had a significantly greater rate of seed failure than did wild-type plants. Our results suggest that ovule fertilization activates *CmACS7* and increases ACC levels, whereas an overexpression of *CmACS7* leads to an increase in ACC content in the ovule prior to fertilization, which can lead to abortion. In conclusion, the present study demonstrated that chestnut ovule abortion is caused by poor fertilization and not by nutritional competition. Optimization of the pollination and fertilization of female flowers is essential for increasing chestnut yield and reducing ovule abortion.

## 1. Introduction

1-Aminocyclopropane-1-carboxylic acid synthase (ACSase) is a key rate-limiting enzyme that can catalyze the synthesis of 1-aminocyclopropane-1-carboxylic acid (ACC) from S-adenosylmethionine (SAM) during ethylene (ETH) biosynthesis, and its activity in plant tissues often determines the rate of ACC and ETH production [1]. Thus, the expression of *ACS* genes can be regulated to effectively control the plant’s growth, development and tolerance of stress [2]. ACS belongs to the family of proteases, and pyridoxal phosphate (PLP) serves as a coenzyme. The structure of the PLP-dependent active center of the enzyme is highly conserved, and the sequence homology of the ACS proteins of various plants ranges from 48% to 97%, with the highest amino acid sequence homology and seven highly conserved regions [3,4]. Of the seven highly conserved regions, the fifth most highly conserved region is considered to be the active center and is involved in the binding of the coenzyme PLP. The C-terminus shows the largest difference and is involved in the regulation of ACS protein degradation [5,6].

Studies on the ACS gene family have focused mainly on model plants such as *Arabidopsis thaliana*, *Solanum lycoperisicum* and *Oryza sativa*, and fewer studies have been conducted on trees. The expression of the ACS gene family exhibits spatiotemporal specificity because the expression levels of *ACS* genes are affected by the plant’s developmental stage, the external environment, and hormonal stimuli [4,7]. *ACS* genes are differentially expressed and exhibit different functions during plant development. Among the 12 *ACS* genes in *A. thaliana*, *AtACS1* is involved in the regulation of leaf senescence; *AtACS2* and AtACS6 induce ETH production under cadmium stress; *AtACS7* is involved in abiotic stress, root growth toward the ground [8,9], and ETH-regulated senescence; and *AtACS8* induces ETH synthesis under Cu^2+^ to improve immunity [10]. The *S. lycoperisicum* genome contains 14 ACS genes; of these, *LeACS1A*, *LeACS2*, *LeACS4* and *LeACS6* are involved mainly in the fruit-ripening process, *LeACS4* plays a crucial role in fruit ripening, and the remaining genes are responsible for ETH synthesis in nutrient-producing organs [11]. A total of 13 homologous ACS genes have been identified in the *Cucurbita maxima* genome; among these, *CmaACS1* and *CmaACS2* play important roles in fruit-specific ripening, *CmaACS3* can increase the number of hermaphroditic buds, shorten the development time, promote the maturation of hermaphroditic flowers and increase the number of fruit sets [12], and *CmaACS7*, which is a sex-determining gene in melons, leads to the development of androdioecious flowers [13]. In poplars, *PtaACS8* is involved in the regulation of plant height and leaf development [14]. In watermelons, *CitACS4* not only is a sex-determining gene but also plays a pleiotropic role in the complete development of female flowers, early fruit development, and the fruit set [15].

Studies have confirmed that the ACC-induced plant response is not caused exclusively by ETH and that ACC may also function as an ETH-independent signaling molecule [16,17]. A comparison of a null (ineffective) mutation in a key component of ETH signaling with the *octuple ACS* (*acs8x*) ETH biosynthesis mutant revealed that ACC, not ETH, is essential for ovule activity because only ACC causes embryo death [10]. In addition, Xu et al. (2008) suggested that ACC may be a signaling molecule in the FEI/SOS5 pathway that regulates cell expansion [18]. In their study of *fei1fei2* mutants, these researchers found that the cell expansion phenotype in roots could be reversed by blocking ETH biosynthesis or 2-aminoisobutyric acid (AIB) but not by disrupting ETH perception either chemically or genetically [19]. Tsang et al. reported that the rapid reduction in primary root elongation in response to short-term cell-wall damage or PAMPs is dependent on ACC biosynthesis and independent of ETH perception [20]. Recent studies have shown that ACC is able to regulate pollen tube orientation independently of ETH signaling and that the abortion of *Arabidopsis* seeds occurs before ovule fertilization [1,21]. These results provide insights into the mechanism of ovule abortion in the chestnut.

*Castanea mollissima*, a species of *Castanea* in Fagaceae that originated in China, is one of the most popular nut species in China. China’s annual production of chestnut nuts ranks first in the world, followed by Spain and Bolivia. However, the yields are relatively low, and ovule abortion is the main cause of the low fruiting rates [22,23]. A correlation has been found between early ovule septation and the size of horn fruits in *Arabidopsis* vinyl mutants [24]. In pumpkin, Li et al. (2021) reported that reduced ETH production or signaling in flowers affects the fruit set and early fruit development [25]. Moreover, ACC is involved in pollen tube orientation independently of ETH signaling, and the absence of ACC leads to *A. thaliana* abortion and seed reduction [23]. In this study, based on the results from transcriptome and metabolome data analyses [22], we performed a genome-wide identification of ACS genes in chestnuts (CmACSs). We then verified the function of key CmACS genes affecting the development of chestnut ovules using a RT–qPCR analysis and genetic transformation of chestnut healing tissues and *S. lycoperisicum* plants. These findings further clarify the influence of the ACS genes on ovule development and, thus, provide a theoretical basis and methodological reference for regulating ovule development and improving the yields of chestnuts.

## 2. Results

### 2.1. Genome-Wide Analysis of the Chestnut ACS Gene (CmACS) Family

Seven CmACS family genes, named CmACS1–CmACS7, were ultimately identified using HMMER software (http://hmmer.org, V3.2.1, CA, USA, accessed on 16 July 2022.) and a bidirectional BLAST comparison (Table 1). The coding DNA sequences (CDSs) of the seven CmACS genes ranged from 1414 to 1607 bp in length, contained 2 to 6 exons, encoded 470 to 535 amino acids, and the encoded proteins had relative molecular weights ranging from 53.14 to 59.25 kDa and isoelectric point (*pI*) values ranging from 5.54 to 8.14. The instability coefficients ranged from 41.07 to 50.79, all of which were greater than 40, which indicated that all the proteins of this gene family in chestnuts were unstable. The subcellular localization prediction of ACS family proteins using the WoLF PSORT website revealed that all of these proteins were localized in the cytoplasm except ACS7, which was localized in the nucleus (Table 1). The signal peptide prediction of the amino acid sequences of the gene family using SignalP 5.0 revealed that none of the CmACS genes carried potential signal peptide sites.

Phosphorylation is a common strategy adopted by higher plants to increase the stability and maintain the activity of ACS proteins. The prediction of potential serine, threonine and tyrosine phosphorylation sites in the CmACS gene family proteins showed that all CmACSs had multiple phosphorylation sites, with the greatest number being present in CmACS7. Among the three types of kinase phosphorylation sites, the most abundant were serines, followed by threonines and then tyrosines (Table S1).

### 2.2. Multiple-Sequence Alignment and Phylogenetic Analysis of the CmACS Gene Family

**Figure 1:**
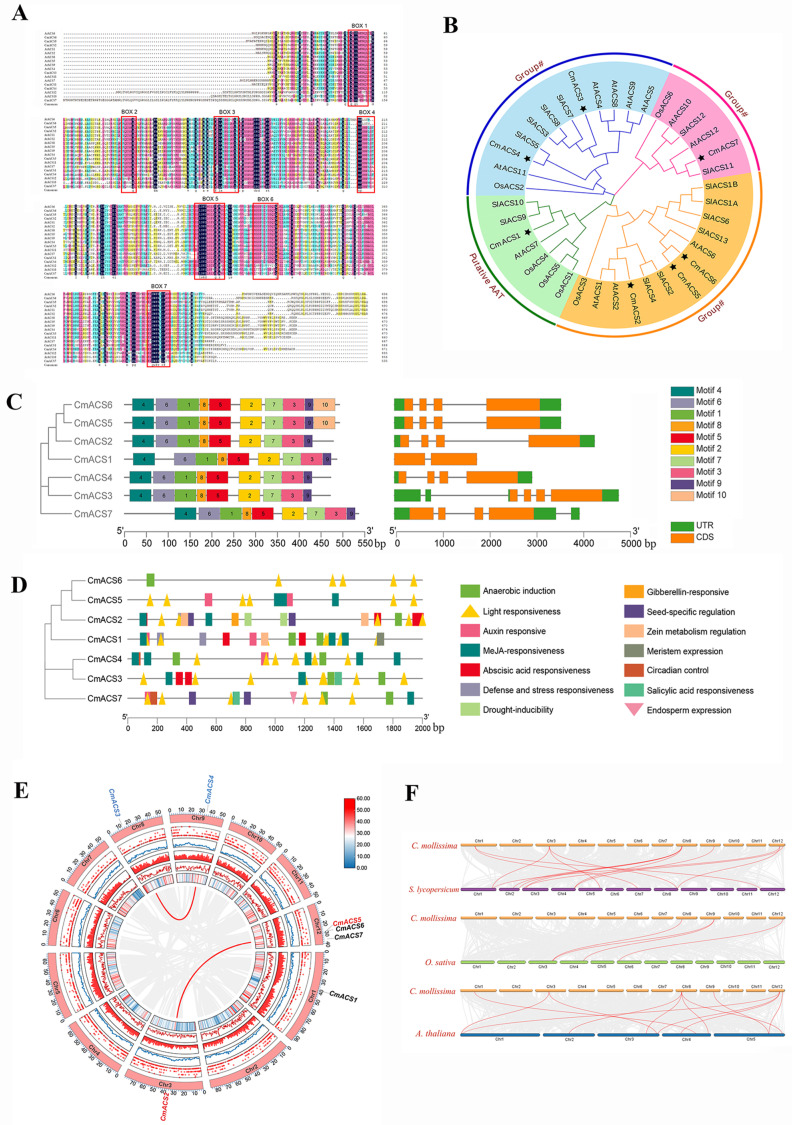
Bioinformatics analysis of the ACS gene family in the chestnut. (**A**) Multiple sequence alignment of the chestnut ACS protein sequences. Boxes 1–7 represent seven conserved structural domains of the ACS gene family. (**B**) Phylogenetic relationships of seven ACSs in chestnut and other plants. A star (★) represents chestnut *ACS* genes. (**C**) Phylogenetic relationships, gene structure, and architecture of conserved protein motifs. Boxes of different colors show different motif. (**D**) Cis-element analysis of the promoters of CmACSs. Cis-elements with similar functions are shown in the same color. Boxes of different colors show different cis-elements. (**E**) Chromosomal distribution of *CmACS* genes. From the outside to the inside, the first circle represents the chromosome coordinates; the second to fifth circles represent the GC content and density of the positive and negative chains, respectively; the sixth circle represents the gene density distribution; and the red lines connect gene pairs. (**F**) Synteny analysis of ACS genes in *C. mollissima*, *S. lycopersicum*, *O. sativa*, and *A. thaliana*. The chestnut genome, protein sequences, and annotation files were searched and downloaded through the GigaDB website (http://gigadb.org/dataset/view/id/100643, accessed on 16 October 2022) [26].

The amino acid sequences of the seven identified chestnut *ACS* gene family members and 12 *A. thaliana* ACS gene family members were subjected to a homology analysis by multiple sequence comparison using DNAMAN software (Version 10.0), and the results revealed that all of the family members possessed seven conserved frames (BOX1–BOX7), as shown in Figure 1A. This finding indicates that the identification of the CmACSs is reliable, and the results of the ACS family’s amino acid sequence comparison also indicate that the CmACS proteins are structurally and compositionally conserved.

To further assess the evolution of the *CmACS* gene family, the full-length protein sequences of 38 ACS proteins from chestnuts, *A. thaliana*, *S. lycoperisicum*, and *O. sativa* were utilized to construct a phylogenetic tree using MEGA-X software (Figure 1B). Based on the differences in the C-terminal sequences, ACSs were categorized into three groups in addition to AtACS10 and AtACS12, which are considered to belong to the aminotransferase type (putative AAT) due to the absence of ACS enzymatic activity. In terms of the ACS gene types, Group I (Figure 1B) contained the most ACS genes (13), which included three chestnut ACS genes, CmACS2, CmACS5 and CmACS6. This group was followed by Group II (Figure 1B), with 11 ACS members and two chestnut ACS members, CmACS3 and CmACS4. The smallest number of ACS members was found in Group III (seven) (Figure 1B), which contained only one chestnut *ACS* gene (*CmACS1*). The putative amino acid transporter (AAT) group (Figure 1B) contained six genes, one of which was a chestnut ACS gene, CmACS7.

### 2.3. Structural Characterization of CmACS Proteins

To study the evolutionary diversity of ACS proteins, the conserved structural domains of the CmACS gene family proteins were analyzed using online MEME software, and a total of 10 motifs were identified, which were named motifs 1 to 10 (Figure 1C; Table S2). All seven CmACS members contained motifs 1 to 9, and only CmACS5 and CmACS6 contained motif 10. The ACS gene family of proteins in the chestnut were found to be highly conserved.

### 2.4. Analysis of Cis-Acting Elements in CmACS Promoters

In this study, the promoter cis-acting elements of the *CmACS* gene family were predicted and analyzed through the PlantCARE website, and this analysis found that the promoters of the *CmACS* gene family mainly contained cis-acting elements related to hormone regulation and responses to stress and light (Figure 1D). All seven *CmACS* genes were predicted to have cis-acting elements related to the response to light, and these elements were the most abundant, followed by hormone response elements and anaerobic-induced elements; these findings suggest that the *CmACS* genes may be involved in the regulation of the plant’s light response. The promoter regions of all the *CmACS* genes except *CmACS6* contained MeJA-responsive elements; *CmACS*1, 4, 5, and 7 contained growth hormone-responsive elements; and *CmACS*1, 2, and 3 contained ABA-responsive elements. Notably, the *CmACS7* gene promoter contained elements related to endosperm expression and seed-specific regulation, suggesting that the *CmACS7* gene may be involved in the regulation of seed development. The *CmACS1* promoter contained elements related to meristematic organization and stress, suggesting that this gene may be involved in the chestnut’s defense and stress responsiveness. These results suggest that members of the *CmACS* gene family may play important roles in pathways such as the light response, adverse stress response, and hormone response pathways, that the *Cm*ACS7 gene may be involved in the regulation of seed development, and that the abnormal expression of this gene may lead directly to chestnut ovule abortion.

### 2.5. Chromosome Localization and Covariance Analysis of CmACSs

An analysis of the chromosomal localization of the *CmACSs* revealed that (Figure 1E) seven *CmACSs* were distributed on five chromosomes of the chestnut; among these, chromosome 12 contained three *CmACSs*, and chromosomes 1, 3, 8, and 9 each had one *CmACS* gene. Covariance analysis revealed that both *CmACS3* and *CmACS4* belonged to Group II, whereas *CmACS2, CmACS5*, and *CmACS6* belonged to Group III. The covariance of the *ACS* genes of the chestnut with those of other species may reflect the phylogenetic relationship of the *ACS* gene family of the chestnut with those of dicotyledonous and monocotyledonous plants. In this study, we constructed comparative covariance maps of chestnut *ACS* genes in two dicotyledonous plants (*A. thaliana* and *S. lycopersicum*) and one monocotyledonous plant (*O. sativa*) (Figure 1F). A total of 13 direct homologous pairs were identified between the chestnut and *S. lycoperisicum ACS* genes, and 4 and 12 direct homologous pairs were identified in *O. sativa* and *Arabidopsis*, respectively, i.e., 7, 3, and 6 *CmACS* genes showed covariance with genes from *S. lycoperisicum*, *O. sativa*, and *A. thaliana*, respectively (Figure 1F). These results indicate that the chestnut is closely related to *S. lycoperisicum*.

### 2.6. Tissue Expression Pattern Analysis of CmACSs

**Figure 2:**
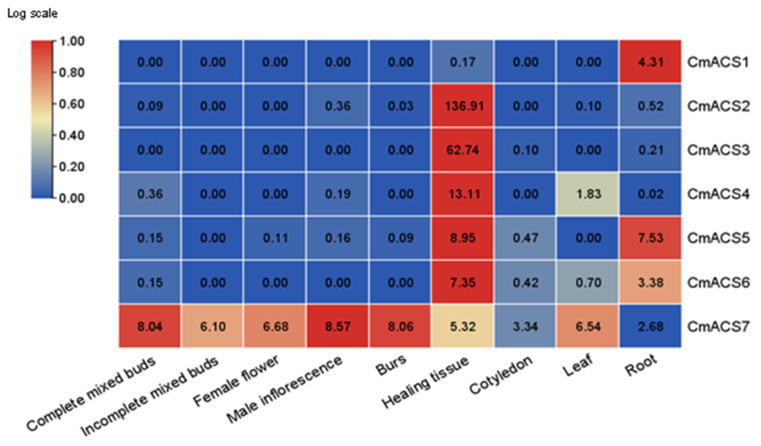
Expression patterns of *CmACS* genes in different tissues. The numbers in the boxes are the relative expressions of the genes.

We performed a qRT–PCR analysis of nine different tissue samples of a chestnut (Figure 2). The results showed that all seven genes were expressed in healing tissues, and the highest expression was observed for *CmACS2*, followed by *CmACS3*, indicating that the genes of this family might be related to cell division, with *CmACS2* playing the largest role. In addition to being more highly expressed in healing tissues, CmACS4 was expressed in mixed and male flowers and leaves, suggesting that this gene may be involved in the regulation of cell division and in the development of leaves and male flowers. *CmACS7* was expressed in all tissues (complete mixed buds, incomplete mixed buds, female flowers, male inflorescences, burs, healing tissues, cotyledons, leaves, and roots); in addition, its highest expression was observed in inflorescences, followed by spiny bracts and fully mixed flowers, and the lowest expression was found in roots, suggesting that the *CmACS7* gene may be involved in the regulation of flower development in chestnuts. *CmACS1* was expressed only in roots and healing tissues, and in roots, the expression of this gene was higher than that of the other genes except *CmACS5*, suggesting that *CmACS1* and *CmACS5* may be associated with cell division in the meristematic zone. Only *CmACS5* and *CmACS7* were expressed in female flowers, and these two genes may play important roles in the growth and development of female flowers.

### 2.7. Expression Analysis of CmACS Genes during Ovule Development

**Figure 3:**
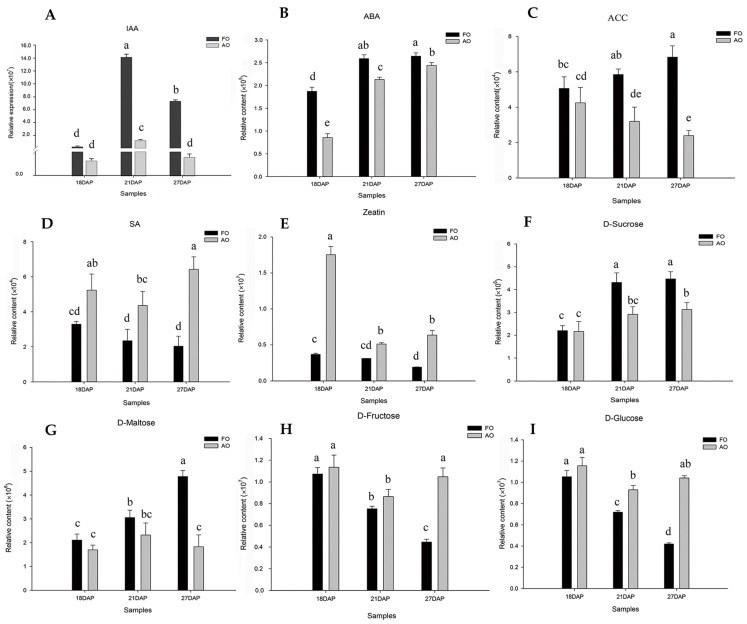
Analysis of *CmACS* gene expression and ACC content at different developmental stages. (**A**) Changes in the expression of *CmACS* genes at different developmental stages in ovules. DAP represents days after pollination, and ovules at 21 DAP and 27 DAP represent fertile ovules after pollination. (**B**–**H**) Relative expression of *CmACS1-7* in fertile and abortive ovules. (**I**) Relative content of ACC in developing and abortive ovules. FO represents a fertile ovule, and AO represents an abortive ovule. Unlabeled lowercase letters in figure are non-significant differences.

To understand the regulatory role of the CmACS gene family members in the processes of pollination and fertilization in chestnuts, we performed a qRT–PCR. The results showed that, except for *CmACS7,* whose expression was significantly elevated, the expression of all the other *CmACS* genes decreased during the critical period of fertilization, suggesting that the chestnut *CmACS7* gene may play an important role in the fertilization process. After the completion of fertilization (18 DAP–21 DAP), the expression of *CmACS*3, *CmACS6*, and *CmACS7* underwent more pronounced changes, with the expression of *CmACS3* and *CmACS6* first increasing from 18 d after pollination (DAP) to 21 DAP and then decreasing at 27 DAP, whereas the expression of *CmACS7* first decreased sharply from 18 DAP to 21 DAP and then decreased slowly to 27 DAP (Figure 3A). These findings indicate that different *CmACS* genes function at different stages of ovule development.

To understand the role of *CmACS* genes in developing and abortive ovules, the expression of *CmACS* genes in fertile and abortive ovules at the same period after pollination was further analyzed (Figure 3B–H), and the results showed that the expression of *CmACS4* was lower in fertile ovules than in abortive ovules in all three periods. The expression of *CmACS1*, *CmACS2*, *CmACS5*, and *CmACS7* was greater in fertile ovules than in abortive ovules at 18 DAP, the expression of *CmACS7* decreased over time after the completion of fertilization, and its expression in abortive ovules was greater than that in fertile ovules at 27 d after the completion of fertilization. The expression of *CmACS7* was greater in abortive ovules than in fertile ovules at 27 d after fertilization. This finding indicates that the *CmACS7* gene plays an important regulatory role in the early stage after fertilization. As the ovules developed after fertilization, the effect gradually weakened. The expression trend obtained for *CmACS7* was similar to that found for *CmACS2*.

The measurement of the ACC content of developing and aborted ovules at 18, 21 and 27 d after fertilization revealed that the ACC content gradually increased during development after fertilization and was greater in fertile ovules than in aborted ovules (Figure 3I), suggesting that the fertilization of ovules induces the expression of *CmACS* genes and promotes the synthesis of ACC. These findings also suggest that chestnut ovule abortion is due to poor fertilization. The joint analysis of the expression and content of *CmACSs* showed that *CmACS7* may play a key role in the fertilization process, whereas, at 21 d after fertilization, *CmACS3* and *CmACS6* play major regulatory roles. After 27 d of development, the expression of *CmACS3* was greater in fertile ovules than in abortive ovules, *CmACS5* was not expressed in either ovule type, and the expression of the remaining genes was lower in fertile ovules than that in abortive ovules. These findings suggest that the functions of *CmACSs* indeed exhibit spatiotemporal specificity, that different family members play regulatory roles at different developmental stages, that *CmACS7* plays a major regulatory role during ovule fertilization, and that its role gradually diminishes after fertilization is completed.

### 2.8. Subcellular Localization

**Figure 4:**
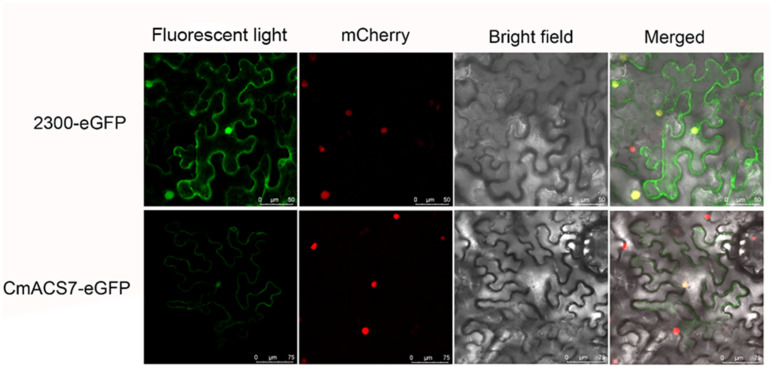
Subcellular localization of CmACS7. The recombinant plasmid (CmAcs7-eGFP) and control plasmid (2300-eGFP) were transiently expressed in tobacco cells. mCherry is a red fluorescent protein variant for nuclear localization that appears in red under fluorescence. eGFP is an enhanced green fluorescent protein that appears in green under fluorescence. The red fluorescence represents the location of the nucleus and the green fluorescence represents the location of the carrier. The scale bars represent 50 μm and 75 μm or the control plasmid and recombinant plasmid, respectively.

The *CmACS7* gene was predicted to localize in the nucleus. To verify the prediction results, the subcellular localization pattern of *CmACS7* was further verified via confocal microscopy (Figure 4). The results showed that the *CmACS7* gene localized not only in the nucleus but also in the cytoplasmic membrane.

### 2.9. Genetic Transformation of Chestnut Healing Tissues

**Figure 5:**
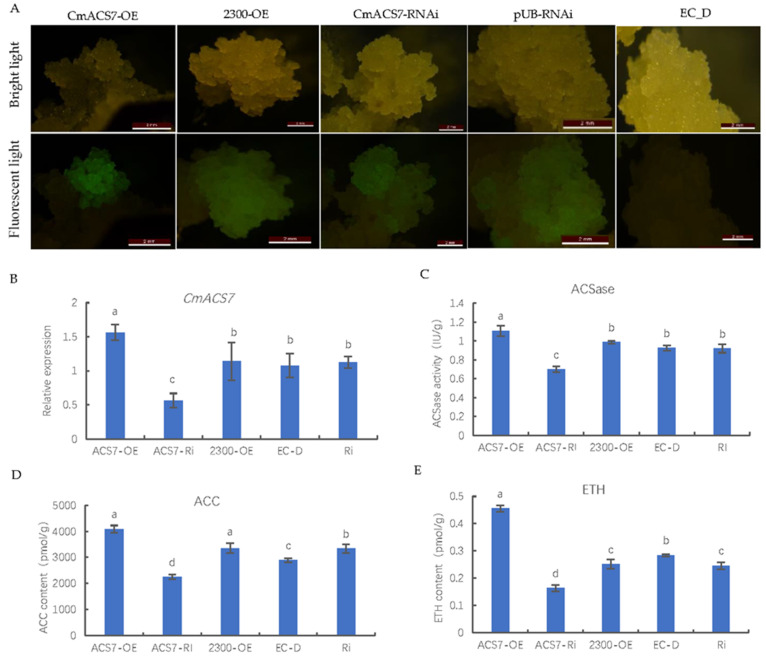
*A. tumefaciens*-mediated transformation of chestnut healing tissues with different vectors. (**A**) Morphology of healing tissues transformed with different vectors under a bright-field and fluorescence microscope at a wavelength of 450–490 nm. CmACS7-OE, CmACS7-RNAi, 2300-OE, and pUB-RNAi represent healing tissues genetically transformed with CmACS7 overexpression vectors, interference vectors, and their empty vectors, respectively; EC_D represents untreated healing tissue. CmACS7-OE is the overexpression vector; 2300-OE is the empty vector of the overexpression vector; the bar represents 2 mm. (**B**) *CmACS7* expression in healing tissues transformed with different vectors. (**C**) ACSase activity. (**D**) ACC content. (**E**) ETH content. Unlabeled lowercase letters in figure are non-significant differences.

To investigate whether *CmACS7* has ACSase activity in the chestnut, chestnut healing tissues containing the overexpression vector *CmACS7*-OE and the double-stranded RNA interference vector *CmACS7*-RNAi were constructed. Fluorescence emission was observed under a fluorescence microscope after 14 days of coculture, and the results are shown in Figure 5A. The fluorescence-carrying healing tissues were selected under a fluorescence microscope and then subjected to screening in a medium containing kanamycin (100 mg·L^−1^) until positive healing tissue was obtained. The qRT–PCR data showed that the CmACS7-OE vector yielded the highest expression in healing tissues, *CmACS7*-Ri resulted in the lowest expression, and the three controls showed intermediate levels of expression and were not significantly different from each other (Figure 5B). We performed a qRT–PCR analysis of all CmACS genes in the genetically transformed positive and WT (EC_D) chestnut healing tissues. The data revealed no significant differences in the expressions of any of the *CmACS* genes except for the *CmACS7* gene. The findings thus demonstrated that the genes’ expression was not disturbed and that RNA interference was specific (Figure S1). The ACSase activity and ACC and ETH contents in healing tissue containing the overexpression vector *CmACS7*-OE and interference vector *CmACS7*-RNAi were determined, and the results are shown in Figure 5C–E. The ACSase activity and ACC and ETH contents were greater in the *CmACS7-*overexpressing healing tissues than in the other healing tissues, whereas the ACC and ETH contents were lower in the *ACS7*-RNAi healing tissues than in the other healing tissues. These findings indicate that *CmACS7* has ACS synthase activity and that the overexpression of *CmACS7* promotes ETH synthesis.

### 2.10. Genetic Transformation of Micro S. lycoperisicum

**Figure 6:**
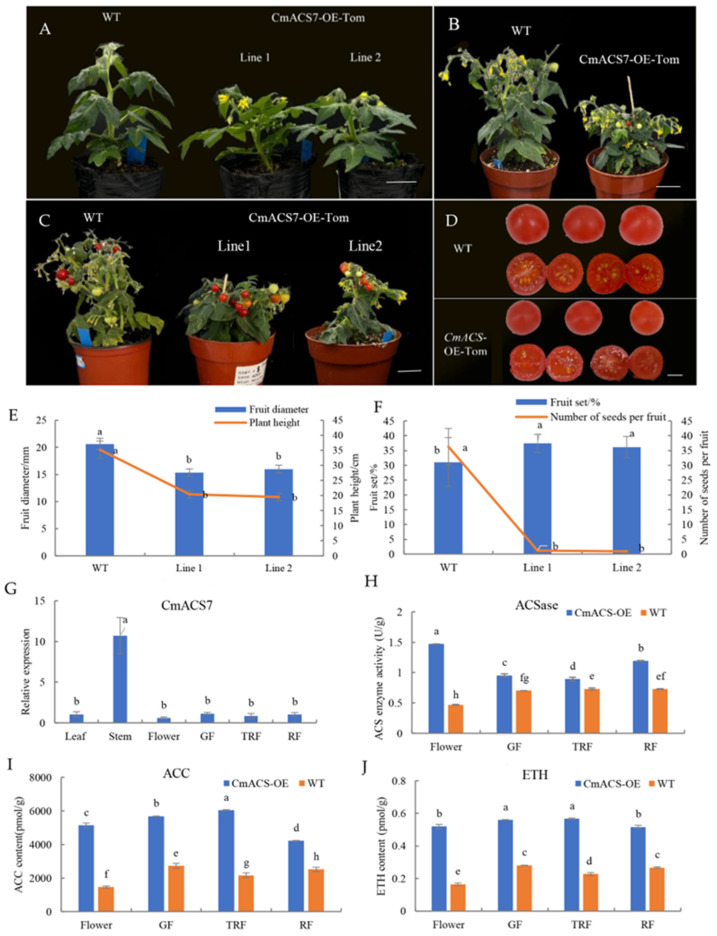
Phenotypic traits of transgenic *S. lycoperisicum* plants overexpressing CmACS7. (**A**) Flower initiation. (**B**) Bloom stage. (**C**) Fruiting stage. (**D**) External phenotypes and seed production of ripe *S. lycoperisicum*es. (**E**) Fruit diameter and plant height of *CmACS7*-overexpressing and WT *S. lycoperisicum* plants. (**F**) Fruit set and number of seeds per fruit of *CmACS7*-overexpressing and WT *S. lycoperisicum* plants. (**G**) Expression of the *CmACS7* gene in different tissues of transgenic *S. lycoperisicum* plants. (**H**) ACSase activity. (**I**) ACC content. (**J**) ETH content. Unlabeled lowercase letters in figure are non-significant differences.

To further investigate the role of the *CmACS7* gene in ovule pollination and fertilization, *CmACS7*-overexpressing transgenic plant lines (*CmACS7*-OE-Tom) were generated using micro *S. lycoperisicum*es. The phenotypic observations of plants with a positive PCR test (Figure S2) showed that, compared to the wild-type (WT) *S. lycoperisicum* plants, the CmACS7-overexpressing plants exhibited dwarfing (Figure 6A,E), early flowering (Figure 6A), early fruit ripening (Figure 6B), and an increased fruit set (Figure 6C,F). However, the CmACS7-overexpressing plants exhibited smaller fruits (Figure 6C,E), a significantly greater rate of abortion, and a significantly lower number of seeds compared to the wild-type plants (Figure 6D,F). Wild-type *S. lycoperisicum*es contain an estimated average of 36.3 seeds per fruit, whereas *S. lycoperisicum*es from overexpression strains have an average of only 1.9 fertile seeds per fruit. The qRT–PCR data showed that *CmACS7* expression was highest in stems, followed by green fruit, and lowest in flowers (Figure 6G). ACSase activity and ACC and ETH contents were measured in the flowers and fruits (green, red, and ripening stages) of the transgenic and wild-type strains. The results showed that the ACC and ETH contents and ACSase activity were greater in the *CmACS7*-OE-Tom strain than in the wild-type strain (Figure 6H–J), which confirmed that *CmACS7* has ACSase activity. The high failure rate of *S. lycoperisicum* plants overexpressing *CmACS7* indicated that the overexpression of the *CmACS7* gene prior to the fertilization of female flowers results in seed abortion.

## 3. Discussion

Ovule abortion is the main cause of a low chestnut yield. By analyzing the transcriptomic and metabolomic data of fertile and aborted ovules of chestnuts, our previous study revealed that *CmACS* genes play important roles in ovule development [22]. In this study, a total of seven ACS family genes were identified in the chestnut genome using various bioinformatic methods. By comparing the amino acid sequences of the seven CmACS gene family members and analyzing the motif and protein structural domains of the protein sequences, we found that this family of genes has a high degree of conservation, approximately 51.36%, that the catalytic domain portion exhibits a high degree of conservation and that the C-terminus exhibits a high degree of variability. All identified proteins were found to have seven conserved boxes (BOX1–7) of the plant ACS protein family, and the proteins of this family all contain aminotransferase class I and II structural domains.

Sequence comparison revealed the presence of the Q98 active site in *S. lycoperisicum* and apples; however, in AtACS10 and AtACS12, this residue was mutated to E and K, respectively, which may be the main reason why putative AAT-type ACSs have ACS enzyme activity in *S. lycoperisicum* and apples, whereas *Arabidopsis* AtACS10 and AtACS12 do not exhibit ACS enzyme activity [1,5]. The same conclusion was reached in a study of *Cucurbita maxima* [12]. An amino acid sequence comparison of the chestnut and *Arabidopsis* proteins revealed that CmACS7, which was clustered with *Arabidopsis* AtACS10 and AtACS12, had a Q98 active site, and the findings indicate that CmACS7 might have ACSase activity. The ACC and ETH contents in healing tissues were examined after the genetic transformation of *CmACS7* into chestnut healing tissues, and the results showed that the ACC and ETH contents and ACSase activity in the healing tissues overexpressing *CmACS7* were greater than those in the control healing tissues, whereas the content in the healing tissues with the interference vector *CmACS7*-Ri was less than that in the controls, which clearly indicates that *CmACS7* has ACS enzyme activity and is involved in ETH synthesis.

Genes can be regulated via interactions between multiple cis- and trans-acting elements in promoter regions [27]. In this study, we predicted and analyzed the promoter cis-acting elements of the chestnut CmACS gene family through the PlantCARE website and found that the promoters of *CmACSs* mainly contained cis-acting elements related to hormone regulation and stress and light responses (Figure 1D). All seven CmACSs were predicted to contain cis-acting elements related to light response, and these elements were the most abundant, suggesting that light influences the expression of *CmACS* genes. The elements with the second and third highest abundance were hormone response-related elements and defense and stress response-related elements. The promoter regions of all *CmACS* genes except *CmACS6* contained MeJA-responsive elements; those of *CmACS1, CmACS4, CmACS5*, and *CmACS*7 contained growth hormone-related elements; and those of CmACS1, CmACS2, and CmACS3 contained ABA-responsive elements. In contrast, the main plant hormones that were found to induce *ACS* genes in previous studies were auxin (IAA), ETH, cytokinin (CK), gibberellins (GAs), and abscisic acid (ABA) [4,28]. This finding is generally consistent with our prediction. In *Arabidopsis* roots, IAA induces the expression of the *AtACS*2, *AtACS4*, *AtACS5*, *AtACS6*, *AtACS8*, and *AtACS11* genes [16]. ABA regulates root growth by modifying the phosphorylation of ACS to regulate the activity of ACS proteins, which affects ETH synthesis [28,29]. In the peach fruit, exogenous jasmonic acid (JA) can increase the transcript abundance of *ACS1*, modulate the ETH-signaling pathway, delay the metabolism of fruit cell membranes, and alleviate cold damage [30]. These hormones are hypothesized to regulate the expression of *CmACS* genes by binding to the corresponding response elements in the promoter region of the genes, thereby affecting plant growth and development and the stress response. Notably, the promoters of *CmACS7* genes contain elements related to endosperm expression and seed-specific regulation. This finding suggests that the *CmACS7* gene may play a role in the regulation of seed development. Furthermore, any abnormal expression of this gene directly impacts the development of chestnut ovules.

Many studies have shown that different genes in the ACS family exhibit spatiotemporal expression specificity and expression differences during growth and development and that the expression of their transcripts is induced by different factors and conditions [31]. In *S. lycoperisicum*, different factors or conditions can induce the expression of different members of the ACS family, and the levels of transcripts of the expressed members exhibit marked differences [32]. The cucumber gene *CsACS2* is expressed only in female flowers during terminal bud development, and its expression is not detected in male flowers [33]. Exogenous ETH treatment can increase *CsACS2* gene mRNA accumulation while suppressing stamen development [33]. In this study, seven *CmACS* genes were differentially expressed in the roots, stems, leaves, and female and male flowers (Figure 2). All *CmACS* genes except *CmACS1* and *CmACS7*exhibited their highest expression in healing tissues. The higher expression of *CmACS1* and *CmACS5* in roots indicated that these two genes were specifically involved in the regulation of root growth and development. Previous studies have also shown that ETH plays an important role in root development, including the formation of lateral and adventitious roots and the regulation of root hair development [20]. Notably, among all *ACS* genes, only *CmACS7* was highly expressed in flowers, suggesting that this gene plays an important regulatory role in the development of chestnut floral organs.

An qRT–PCR analysis of chestnut ovules at different developmental stages found that *CmACS7* was significantly upregulated before and after fertilization (15–18 DAP); at 18 DAP, the expression of *CmACS7* was greater in fertilized ovules than in unfertilized ovules, suggesting that fertilization induces the expression of this gene. Measurements of the ACC content in fertile and aborted ovules at the three stages revealed that the ACC content in fertile ovules was greater than that in aborted ovules, which indicated that chestnut ovule abortion was due to fertilization failure. By counting the fertile ovules of chestnuts after fertilization, our research team revealed that ovule abortion mainly occurs at the early stage after the completion of ovule fertilization [34,35], which indicates that the main reason for the low chestnut yield is poor fertilization.

Currently, research on *ACS* gene function has focused on areas related to adverse stress responses and fruit ripening, whereas fewer studies have investigated seed development. Oeller et al. (1991) transferred the cDNA antisense sequence of ACC synthase into *S. lycoperisicum* and succeeded in obtaining ripening-impaired transgenic *S. lycoperisicum* plants, which showed delayed ripening [36]. Using the phenomenon of homologous coinhibition to control endogenous ETH synthesis, the coinhibitory transgene can downregulate the expression of the ACS gene and thus delay the flowering time in pineapples [37]. In our study, we found that *CmACS7* gene-overexpressing small *S. lycoperisicum* lines exhibited early flowering and early fruit ripening, which is consistent with the findings of previous studies. In addition, we found that the overexpression *S. lycoperisicum* lines exhibited a significantly greater rate of seed abortion than did the wild-type lines. For *Arabidopsis thaliana*, Mou et al. (2020) found that ACC signaling in ovules is involved in the induction of pollen tube rotation and the efficient delivery of pollen and that the number of seeds nearly doubles in the presence of ACC [21]. These findings appear to contradict those obtained in our study. The reason for this difference may be that the effect of ACC on the regulation of pollen tubes occurs at specific pollination and fertilization periods, which can lead to seed abortion if high ACC expression persists during other periods. Based on these results, we hypothesized that there is a dose effect of ACC on pollen tube attraction, and a too-high concentration of ACC not only fails to induce pollen tubes to enter the ovule for fertilization but also disrupts their orientation. This hypothesis effectively explains the higher ACC content in fertilized ovules compared to aborted ovules. Fertilization triggers the upregulation of the *CmACS7* gene, leading to increased ACC levels that inhibit polyspermy. In contrast, unfertilized ovules maintain lower ACC levels to attract pollen tubes for successful fertilization. Of course, it cannot be ruled out that the different modes of regulation are due to species differences. This can be further verified by pollen tube experiments in the future.

In summary, the members of the *CmACS* family play important regulatory roles in the development of chestnut ovules, especially the *CmACS7* gene, which directly affects the normal development of ovules. The ACC content of fertile ovules was greater than that of unfertilized ovules, indicating that fertilization induced the expression of the *CmACS7* gene, and the increase in the ACC content after fertilization was key to ensuring normal ovule development. Overexpression of the *CmACS7* gene in small *S. lycoperisicum* resulted in seed abortion, indicating that the overexpression of *CmACS7* before fertilization leads to aborted ovules. The ACC content in developing ovules was greater than that in aborted ovules, suggesting that chestnut abortion is due to unfertilized ovules rather than nutrient competition. Therefore, ensuring complete fertilization constitutes an important strategy for reducing the rate of chestnut ovule abortion and increasing chestnut yield.

## 4. Materials and Methods

### 4.1. Plant Materials

The test material was obtained from the National Chestnut Seed Breeding Base at Weijinhe, Zunhua City, Hebei Province (117°45′11″ E, 40°21′22″ N). The test mother plant was ‘Zunhua Duanci’ (*C. mollissima* ‘Zunhua Duanci’); the pollinated varieties were ‘Zibo’ (*C. mollissima* ‘Zibo’) and ‘Dongling Mingzhu’ (*C. mollissima* ‘Dongling Mingzhu’); and the age of the tree was 25 years. During the flowering period, the plants were subjected to bagging without pollination or manual full pollination, 10 trees were subjected to each treatment, and 50 involucres were selected from each tree. Please refer to Cui et al. for the specific procedure [22].

### 4.2. Identification and Protein Structure Analysis of the CmACSs

The chestnut genome, protein sequences, and annotation files were searched and downloaded through the GigaDB website (http://gigadb.org/dataset/view/id/100643, Beijing, China, accessed on 16 July 2022); the *A. thaliana* whole genome, protein sequences, and gene annotation files were downloaded through TAIR (https://www.arabidopsis.org/, TAIR10/Araport11 version, accessed on 16 July 2022); the *O. sativa* whole genome, protein sequences, and annotation files were obtained through the *O. sativa* database (https://www.ricedata.cn/gene/, IRGSP-1.0 version, accessed on 16 July 2022); and the *S. lycoperisicum* whole genome, protein sequences, and annotation files were obtained through the Sol Genomics Network (https://solgenomics.net/organism/solanum_lycopersicum/genome, Genome version SL4.0 and Annotation ITAG4.0, accessed on 16 July 2022). To ensure that the identified gene family members were accurate, we used both conserved structural domain prediction (Hidden Markov models (HMMs) and HMMER search) and a homologous sequence comparison via BLAST for identification. First, the candidate ACS genes of the chestnut were screened according to the sequence of the *A. thaliana* ACS gene family using the bidirectional BLAST function of TBtools software [38] (https://github.com/CJ-Chen/TBtools/releases, version 2.069, Guangzhou China, accessed on 19 March 2021). Second, the conserved domains of the candidate genes were identified using the SEQUENCE SEARCH tool (http://pfam.xfam.org/search/sequence, accessed on 17 July 2022) in the online analysis software Pfam, which showed that the proteins containing the conserved domains of aminotransferase classes I and II were members of the ACS family. The structural domains of the ACS proteins were predicted with the NCBI-CDD database (https://www.ncbi.nlm.nih.gov/Structure/bwrpsb/bwrpsb.cgi, accessed on 17 July 2022) and the SMART website (http://smart.embl-heidelberg.de/, accessed on 17 July 2022) [39], eliminating sequences that did not contain transaminase I and II protein structural domains. The physicochemical properties of the CmACS gene family (CmACSs) were analyzed using the ExPASy website (https://web.expasy.org/compute_pi/, accessed on 18 July 2022). The presence or absence of signal peptides and their sites for ACS gene family proteins were predicted using the online server SignalP 5.0 (http://www.cbs.dtu.dk/services/SignalP/, accessed on 18 July 2022). The sites of phosphorylation in the chestnut ACS proteins were predicted using the NetPhos 3.1 server (http://www.cbs.dtu.dk/services/NetPhos/, accessed on 18 July 2022). The subcellular localization of CmACS family proteins was predicted using the WoLF PSORT webpage online tool (https://wolfpsort.hgc.jp/, accessed on 18 July 2022).

The conserved motifs of the ACS protein sequences were predicted using the MEME website (http://meme-suite.org/tools/meme, accessed on 18 July 2022), and the maximum number of motifs was set to 15 to identify the conserved protein motifs of the gene family members. The structures of the genes were visualized using the TBtools visualization tool in combination with genome annotation files.

### 4.3. Analysis of Cis-Acting Elements of ACS Promoters

The 2000-bp sequence upstream of the transcription start site of the *CmACS* genes was extracted as the promoter region using TBtools software and the PlantCARE website (http://bioinformatics.psb.ugent.be/webtools/plantcare/html/, accessed on 19 July 2022) was used to predict and analyze the ACS family genes. The cis-acting elements of the promoter region were predicted and analyzed, and the promoter position was then visualized using the TBtools tool.

### 4.4. Multiple-Sequence Comparison and Construction of the Phylogenetic Evolutionary Tree of CmACSs

The ACS protein sequences of four species, chestnut, *A. thaliana*, *S. lycoperisicum*, and *O. sativa*, were compared using DNAMAN software (https://www.lynnon.com/, Version 9.0, CA, USA, accessed on 19 July 2022), and a phylogenetic evolutionary tree was constructed using the neighbor-joining method. The evolutionary tree was constructed using the online website iTOL (https://itol.embl.de/, accessed on 19 July 2022) to analyze the evolutionary relationships between the ACS family genes of the chestnut and those of other species.

### 4.5. Chromosome Location and Collinearity Analysis

Detailed chromosomal locations of the *CmACS* genes were obtained from the GFF genome files downloaded from the database and their predicted positions on the chromosomes were mapped using TBtools software, with the relative positions indicated by the gene names. Homology maps of ACS homologs between chestnut *A. thaliana*, *S. lycoperisicum* and *O. sativa* were obtained using TBtools software.

### 4.6. Analysis of the Expression of CmACSs in Different Tissues

The plant materials were obtained from 10-year-old *C. mollissima cv*. Zunhua Duanci at the National Chestnut Seed Breeding Base, Weijinhe, Zunhua City, Hebei Province (117°45′11″ E, 40°21′22″ N). Chestnut burs, completely mixed flower buds, incompletely mixed flower buds, leaves, female flowers, male inflorescences, cotyledons, roots, and healing tissues from laboratory-preserved samples were collected, rapidly frozen in liquid nitrogen, and stored in an ultralow-temperature freezer at −80 °C for subsequent qRT–PCR analysis.

The total RNA from each tissue sample was extracted using an Omega Bio-Tek@ Plant RNA Kit (R6827) (Omega, New York, NY, USA) and used for the reverse transcription of cDNA after the concentration and quality were determined with an ultramicro spectrophotometer (Thermo NanoDrop 2000, Waltham, MA, USA). The reverse transcription of cDNA was performed using a HiScript III 1st Strand cDNA Synthesis Kit (+gDNA wiper) (Vazyme, Nanjing, China). ChamQ Universal SYBR qPCR Master Mix (Vazyme, Nanjing, China) was used for qRT–PCR. The relative expression levels were calculated using the 2^−∆∆CT^ formula, and three biological replicates of the experiment were performed. The *CmActin* gene of *C. mollissima* was selected as the reference gene; the primers used are shown in the Supplementary Materials (Table S3). Specific reaction programs were set up according to the reference instruction manual.

### 4.7. Expression Analysis of CmACSs during Ovule Development

Previous studies have shown that chestnut ovules complete fertilization at 18 DAP [35]. Using 18 DAP as the reference time point, ovules at 7, 15, 18, 21, and 27 DAP and bagged unpollinated ovules (18WF, collected at the same time as the 18 DAP ovules) were collected as experimental materials after full artificial pollination. The ovules collected at 7 and 15 DAP were considered to be prepollinated and prefertilized ovules, respectively, and those collected at 18, 21 and 27 DAP were postfertilization ovules. At 21 DAP, fertile ovules and abortive ovules could be distinguished from each other by their appearance; therefore, the ovules collected at 21 and 27 DAP were further divided into fertile ovules (21FO, 27FO) and abortive ovules (21AO, 27AO). For details, please refer to Cui et al. (2022) [23]. The stripped ovule samples were all packed in 1.5 mL freezing tubes, and three replicates of each sample were included, resulting in a total of 24 samples. The test materials were frozen in liquid nitrogen after peeling and stored in an ultralow-temperature freezer at −80 °C. qRT–PCR was performed as described in Section 2.5.

### 4.8. Construction of CmACS7 Gene Overexpression and Interference Vectors

Vectors were constructed by homologous recombination. The plant overexpression vector used was pCAMBIA2300, and the interference vector used was pUB-GFP-RNAi-Kan. ACS7-PCAMBIA2300-GFP was double digested with *Sac*I and *Xba*I. The primers used in this study were designed according to the sequence of the *CmACS7* gene, and the primers used for the construction of the overexpression vectors are shown in Table S3. The pUB-GFP-RNAi-Kan interference vector was constructed by the double digestion of both the positive- and negative-sense strands. The primers used for silencing vector construction are shown in Table S3.

The ClonExpressR Ultra One Step Cloning Kit C115 (Vazyme Biotech Co., Ltd., Nanjing, China) was used for the homologous recombination of the linearized vector and target fragment, and transformed *Escherichia coli DH5α* (Weidibio, Shanghai, China) was plated on a solid LB medium supplemented with 50 mL·g^−1^ of kanamycin (Kan). Single colonies were picked for PCR, and the colonies that were initially identified as positive were sent to the Sequencing Department of Sangon Biotech (Beijing, China) for sequencing verification. The constructed vectors *CmACS7*-pCAMBIA2300 and *CmACS7*-pUB-GFP-RNAi-Kan and the vectors pCAMBIA2300 and pUB-GFP-RNAi-Kan without *CmACS7* were transformed into *Agrobacterium tumefaciens* GV3101 competent cells (Weidibio, Shanghai, China) via freeze–thawing, and the positive clones were identified and screened. The samples were stored at −80 °C for subsequent experiments. The PCR primers used are shown in Table S3.

### 4.9. Subcellular Localization

A transient transformation system using tobacco epidermal cells was used to evaluate the subcellular localization of selected *ACS* genes in chestnut. The recombinant vector *CmACS7*-pCAMBIA2300 was transformed into *A. tumefaciens* strain GV3101 competent cells [40]. Four-week-old tobacco (*Nicotiana benthamiana*) plants with the m-cherry marker were selected for cell infiltration. The GFP fluorescence in the transformed leaves was visualized using a BX 63 automated fluorescence microscope (OLYMPUS, Tokyo, Japan).

### 4.10. Genetic Transformation of Chestnut Healing Tissues

Chestnut healing tissues were transformed using the constructed *A. rhizogenes* strains containing the *CmACS7*-pCAMBIA2300 (*CmACS7*-OE) and *CmACS7*-pUB-GFP-RNAi-Kan (*CmACS7*-Ri) plasmids, and the corresponding plasmids without the *CmACS7* gene were used as controls. The method used for the genetic transformation of chestnut healing tissues was previously described by Yu et al. [41]. After 14 days of coculture following the infiltration period, the expression of eGFP was observed under an Axiocam 506 color fluorescence microscope (ZEISS, Oberkochen, Germany). The fluorescence-carrying healing tissues were identified as positive for healing and selected for a further expansion culture via resistance screening (with 100 mg·L^−1^ kanamycin). A qRT–PCR of the healing-positive tissues was performed to measure gene expression. Enzyme-linked immunosorbent assay (ELISA) kits were used to determine the ACC content, ETH content and ACSase activity in healing tissues with different plasmids, and the specific experimental procedures were performed as described in the instruction manual.

### 4.11. Transformation of S. lycoperisicum by A. tumefaciens Containing CmACS7

The overexpression vector *CmACS7*-pCAMBIA2300, constructed as described above, was used for the genetic transformation of micro*S. lycoperisicum* plants, which was performed according to the method described by Cortina and Culiáñez-Macià (2004) through the *A. rhizogenes* infestation of *S. lycoperisicum* cotyledons [42]. Adventitious shoots from infested *S. lycoperisicum* plants were first selected as transformed lines on MS media (containing kanamycin 50 mg·L^−1^), after which the resulting rooted explants were planted in pots. Leaf DNA from transgenic and wild-type *S. lycoperisicum* plants was extracted with a DNAsecure Novel Plant Tissue DNA Extraction Kit (Tiangen, Beijing, China) for identification.

The expression of the positive plants identified by PCR was analyzed (Figure S1). RNA was extracted from the stems, leaves, flowers and fruits of the positive control plants, and the qRT–PCR analysis was performed as described in Section 2.5. A phenotypic analysis of the identified positive transgenic *S. lycoperisicum* plants was performed. Ten plants were selected, the plant height and fruit set were measured, 30 fruits were randomly selected, the fruit diameter was measured, and the number of seeds per fruit was counted. The ACC content, ETH content, and ACSase activity in flowers and fruits (green fruits, GFs; trans-red fruits, TRFs; Red fruits, i.e., mature fruits, RFs) were measured.

## 5. Conclusions

In this study, seven members of the CmACS gene family were identified from the chestnut genome and analyzed bioinformatically. Via qRT–PCR, we demonstrated the spatiotemporal and temporal expression specificity of the CmACSs and showed that these proteins play important roles in ovule fertilization. The ACC content in fertile ovules was greater than that in unfertilized and aborted ovules, indicating that ovule abortion at this stage was due to unfertilized ovules. The genetic transformation of chestnut healing tissues confirmed that CmACS7 has ACS enzyme activity, although it clusters with the AtACS10 and AtACS12 of Arabidopsis (AtACS10 and AtACS12 were identified to have no ACS enzyme activity). The genetic transformation of micro-*S. lycoperisicumes* further clarified the role of the *CmACS7* gene in pollination, fertilization and ovule development. After a series of experiments, we drew the following important conclusion: ovule fertilization induces the expression of the *CmACS7* gene and increases the ACC content, and overexpression of the *CmACS7* gene prior to fertilization leads to poor fertilization, which in turn causes ovule abortion. Therefore, ensuring the complete pollination and fertilization of female flowers is an important strategy for improving chestnut yield and reducing ovule failure.

## Figures and Tables

**Table 1:** Characteristics of the seven ACSs in chestnuts.

Gene Name	Gene ID	Subcellular Localization	CDS Length/bp	No. of Exons	Protein Characteristics				Signal Peptide Sites
					Amino Acid Length/aa	Instability Coefficient	Molecular Weight/kDa	Isoelectric Point	
*CmACS1*	*Cm01G01961*	Cytosol	1459	2	485	50.07	54.60	6.06	No
*CmACS2*	*Cm03G01038*	Cytosol	1435	4	477	45.94	53.82	8.14	No
*CmACS3*	*Cm08G00844*	Nucleus	1414	6	470	41.07	53.25	6.90	No
*CmACS4*	*Cm09G01545*	Cytosol	1417	4	471	43.91	53.14	5.54	No
*CmACS5*	*Cm12G01476*	Cytosol	1477	4	491	43.43	55.17	6.74	No
*CmACS6*	*Cm12G01477*	Cytosol	1477	4	491	43.43	55.17	6.74	No
*CmACS7*	*Cm12G01910*	Nucleus	1607	5	535	50.79	59.25	7.02	No

## Data Availability

Data are contained within the article.

## Supplementary Materials

The following supporting information can be downloaded from https://www.mdpi.com/article/10.3390/ijms25084454/s1.
