# Supplementary material for: Genome-Wide Characterization and Functional Validation of the ACS Gene Family in the Chestnut Reveals Its Regulatory Role in Ovule Development
Source: Int J Mol Sci. 2024 Apr 18;25(8):4454. doi: 10.3390/ijms25084454 (PMC11049808; doi:10.3390/ijms25084454)
Supplement: Supplementary file 1 [file ijms-25-04454-s001.zip › suppl.figure S2 .pdf]

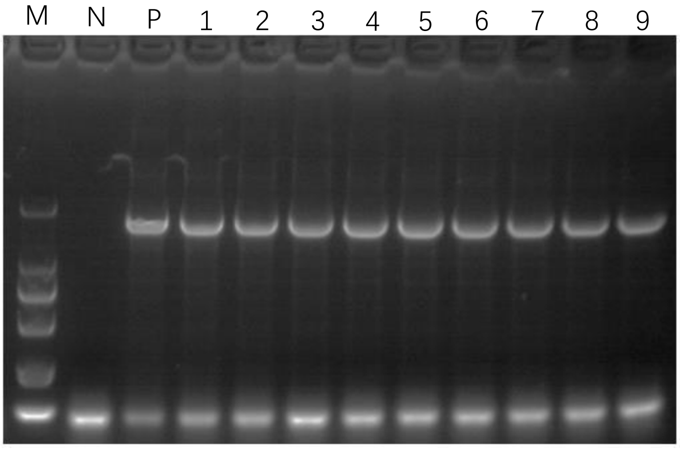

Figure S2. PCR identification of positive tomato plant with *CmACS7* gene

Note: Size of the target band:1629bp; M,marker, from top to bottom is 2000bp,1000bp,750bp; N, negative; P, positive; 1-9, Positive transgenic tomato lines
