# Supplementary material for: Genome-Wide Characterization and Functional Validation of the ACS Gene Family in the Chestnut Reveals Its Regulatory Role in Ovule Development
Source: Int J Mol Sci. 2024 Apr 18;25(8):4454. doi: 10.3390/ijms25084454 (PMC11049808; doi:10.3390/ijms25084454)
Supplement: Supplementary file 1 [file ijms-25-04454-s001.zip › suppl.Figure.S1.pdf]

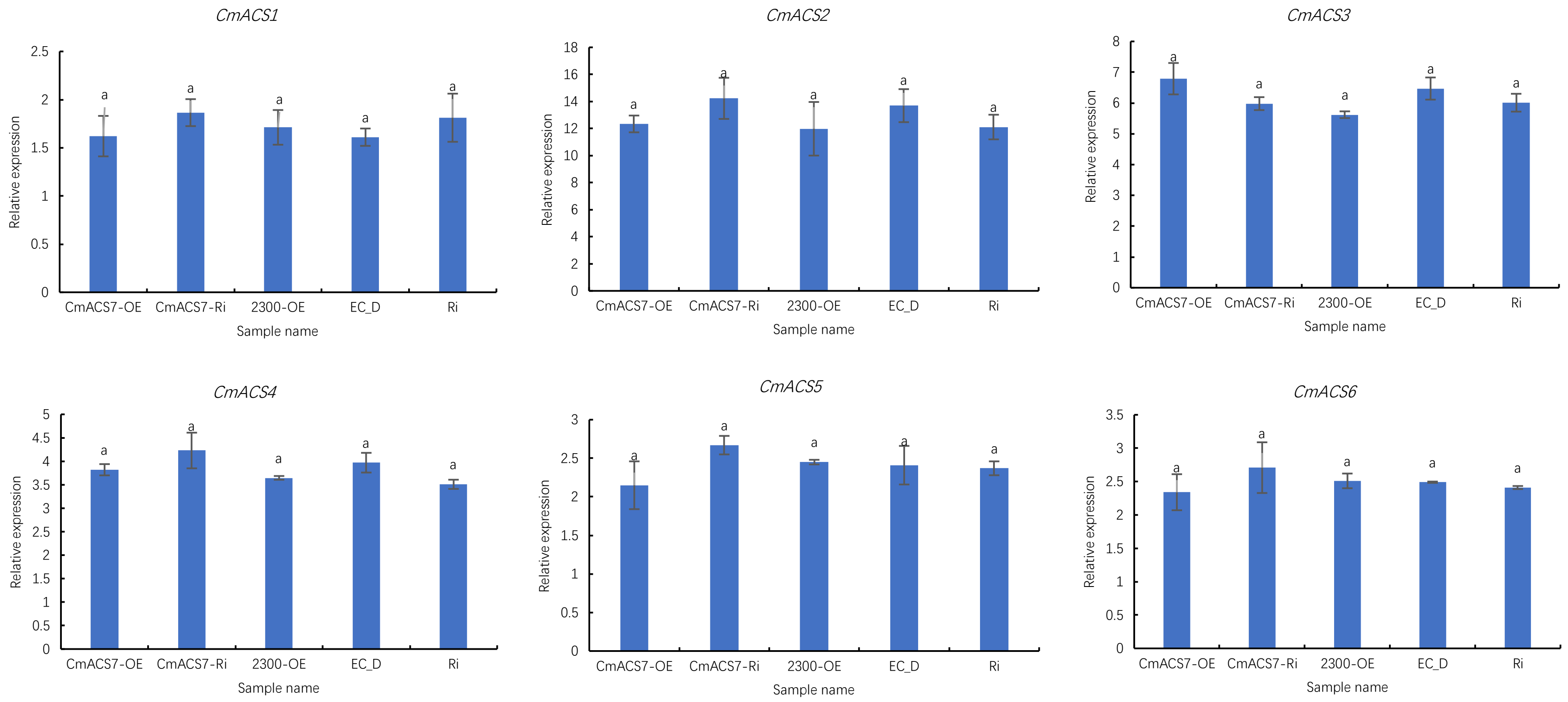

Figure S1. Chestnut *CmACS* gene expression in different genetically transformed healing tissues.

CmACS7-OE, CmACS7-Ri, 2300-OE, and Ri are represent the healing tissues of genetically transformed with *CmACS7* overexpression vectors, interference vectors, and their empty vectors, respectively; EC_D is represents untreated healing tissue.
